# Gene Expression Study in Gilthead Seabream (*Sparus aurata*): Effects of Dietary Supplementation with Olive Oil Polyphenols on Immunity, Metabolic, and Oxidative Stress Pathways

**DOI:** 10.3390/ijms252212185

**Published:** 2024-11-13

**Authors:** Martina Torricelli, Andrea Felici, Raffaella Branciari, Massimo Trabalza-Marinucci, Roberta Galarini, Massimo Biagetti, Amedeo Manfrin, Laura Boriani, Eleonora Radicchi, Carla Sebastiani, Marcella Ciullo, David Ranucci, Francesco Agnetti

**Affiliations:** 1 Istituto Zooprofilattico Sperimentale dell’Umbria e delle Marche “Togo Rosati”, 06126 Perugia, Italy; a.felici@izsum.it (A.F.); r.galarini@izsum.it (R.G.); m.biagetti@izsum.it (M.B.); l.boriani@izsum.it (L.B.); e.radicchi@izsum.it (E.R.); c.sebastiani@izsum.it (C.S.); m.ciullo@izsum.it (M.C.); f.agnetti@izsum.it (F.A.); 2 Department of Veterinary Medicine, University of Perugia, Via San Costanzo 4, 06126 Perugia, Italy; massimo.trabalzamarinucci@unipg.it (M.T.-M.); david.ranucci@unipg.it (D.R.); 3 Istituto Zooprofilattico Sperimentale delle Venezie, Viale Dell’Università, 10, 35020 Legnaro, Italy; amanfrin@izsvenezie.it

**Keywords:** gilthead seabream (*Sparus aurata*), nutrigenomic, polyphenols, circular economy, sustainability, gene expression, metabolic pathway, immunity pathway, oxidative stress pathway

## Abstract

In an era with an ever-growing population, sustainability and green transition are the main milestones to be considered within the current European Green Deal program, and the recovery of by-products for the integration of feed with bioactive molecules, that are sustainable and with high nutritional value, is an ambitious mission to be explored also in aquaculture. Olive oil extraction produces a range of solid and liquid by-products, in varying proportions depending on the utilized production techniques, all of which are considered as possible pollutants. However, these products are also rich of polyphenols, bioactive molecules with several and well-known beneficial properties (antimicrobic, anti-inflammatory, antioxidant, and immune-modulating). On this basis, this work aimed at evaluating the effects of dietary supplementation with polyphenols derived from olive mill wastewater on growth performance and on gene expression modulation, by means of RT-qPCR assays, in farmed *Sparus aurata*. Particularly, some target genes of metabolic, immunity, and oxidative stress pathways have been investigated in breeding gilthead seabream. Differential gene expression analysis was carried out, and differences between the control group (n = 9) and the treated one (n = 9) were computed with Student’s t test. The results have highlighted that supplemented feed enhanced fish growth, with a significant feed conversion ratio between the two groups. Furthermore, the polyphenol diet had a beneficial impact on gene expression fold with a level of significance for *fatty acid binding protein 2*, *superoxide dismutase 1*, and *interleukin-12* genes at hepatic or intestinal district. These significant and promising preliminary findings promote, in the future, other investigations on polyphenolic by-products and on their putative or possible re-utilization in fish feeding.

## 1. Introduction

Every year in the European Union (EU), 88 million tons of food waste are generated, mostly from un-recycled raw materials and the food industry, with associated costs estimated at about EUR 143 billion. Food industry by-products have a huge environmental impact, being responsible for 8–10% of global and about 6% of total EU greenhouse gas emissions, pollution, and waste production [1]. The circular bio-economy and the green transition, in line with the current European model of Green Deal [2], are intended to reuse, repair, and recycle by-products, achieving sustainable consumption and production, with a consequent reduction in impactful environmental damages [3]. This concept should be applied also for the generation of innovative and sustainable feeds for livestock, including local aquaculture, an always fast-growing production sector [4]. Particularly, aquaculture is remarkably one of the most promising in the world, among the food-producing industries, due to the impactful increase over the past two decades [5]. Indeed, fish, high in protein and in polyunsaturated fatty acids as well as rich of omega 3 and vitamins (such as vitamin B2 and B6), represents an alternative nutritional animal-derived food source for a global population in constant growth, having proven their beneficial health effects [6,7,8].

In addition, it is estimated that the human population will grow up to 9.7 billion by 2050, determining a 25–70% challenging increase in the supply of high-quality, nutrient-rich food [9]. Considering that by-products, besides being rich in potentially useful compounds, are considered industrial waste with a high environmental impact, in recent years they have been object of different studies on their possible treatment and reuse, also in the livestock sector [10,11]. Some bioactive molecules, as phytonutrients, exert a protective action particularly against the oxidative stress state and, therefore, on the welfare conditions of the animals, including a better stability of the ecosystem and the intestinal microbiota. Additionally, they allow improvement of the qualitative characteristics of the derived products, both from the hygienic and from a nutraceutical point of view, with a recorded increase in anti-microbial activity [12,13,14]. In fact, the use of additives of natural origin could also counteract the massive use of antibiotics to contain techno-pathologies, helping to reduce the risk of antibiotic resistance phenomenon, which is becoming increasingly impactful and widespread in intensive livestock farming as well as in the aquaculture sector, up to humans [15,16]. Furthermore, improved and alternative fish nutrition could reduce feed waste, resulting in enhanced financial sustainability [17].

On this basis, the main purpose of this research was to evaluate the effects of a diet supplemented with polyphenol extract obtained from olive mill waste waters—OMWW in gilthead sea bream farmed in central Italy at one of Tyrrhenian coast. Gilthead seabream is one of the most commercially valuable marine fish species in Mediterranean aquaculture but also in Northern European countries. The worldwide production has reached 252,406 metric tons in 2019 [18], representing 4.1% relative to the production of the primary aquaculture species, at a rather low cost [19]. Alongside the evaluation of the growth parameters, the bio-molecular workflow was based on RT-qPCR assays, here developed, in order to assess the relative gene expression level of some genetic targets representative of the metabolic pathway, immune–inflammatory processes, and the oxidative stress response. The differential gene expression was assessed on fish control group and on the treated one, in order to evaluate the effects of polyphenol molecules able to exert an antioxidant, an anti-inflammatory and an antibacterial effect [20,21,22,23]. This preliminary work wants to fill some gaps derived from the limited literature data about the reuse, in the aquaculture sector, of natural substance waste as feed supplements and about the effect exerted by these olive oil by-products on the organism, through a “nutrigenomic approach”.

## 2. Results

The growth index and other evaluation parameters as well as the gene expression study were performed on a control group of nine gilthead seabream (*Sparus aurata*) fed with a conventional feed and on a group of other nine gilthead seabream (*Sparus aurata*) treated with diet with a specific concentration level of polyphenolic supplementation feed.

### 2.1. Polyphenolic Compounds Chemically Characterized

Regarding polyphenolic extract, the contribution in terms of concentration (mg/g) of the sum of vanillic acid and luteolin is comparable to that of tyrosol (Table 1). However, hydroxytyrosol is still the most abundant polyphenol among those determined with a hydroxytyrosol/tyrosol ratio of around 5.5. On the other hand, in the supplemented feeds, the ratio of hydroxytyrosol to tyrosol decreases (<2) due to the natural presence of the latter in the feed constituents. Indeed, in supplements and in supplemented feeds, the main polyphenolic components are hydroxytyrosol and tyrosol, characteristic of the olive plant, followed by vanillic acid and vanillin (Table 2).

In Table 2, the concentration (mg/kg) of the detected analytes compared to the total polyphenols (mg/kg), both in the supplemented feeds and in the control feed, is reported.

### 2.2. Health Status and Weight Indicator of Gilthead Seabram Fed Wit Integrated Diet

Mortality was not recorded in any period of the study and no positivity for bacterial, viral, and parasitic pathogens was registered, highlighting fish general health conditions, particularly for the treated specimens.

Histopathological examination of the liver and intestine was also carried out and no differences or abnormal appearance, as tissue lesions, both in the treated and control group, was observed.

As shown in Supplementary Table S1 a significant improvement of the feed conversion ratio was recorded, in particular 1.85 and 1.45, respectively, for the control group and for the treated one. In fact, on the basis of the potential growth-promoting effect of polyphenols [24], fish fed with the experimental diet showed an effective increase in weight. In the intermediate measurement, a higher (117.1%) weight gain in the supplemented group, when compared to 81.8% of the control group, was observed. In addition, a significantly higher weight increase was recorded at the end of the experiment (176.9% in the supplemented group respect to 124.7% in the control group). A similar trend was observed for the specific growth rate.

### 2.3. Molecular Assays Optimization

RNAs extracted from the liver and posterior intestine were of good quantity (at about an average of 4 µg/µL) and quality considering the 260/280 and the 260/230 ratios.

Regarding the comparison between the literature and *in-house* designed systems, at the same amplification conditions, for *fatty acid binding protein 2* (*FABP2*), *alkaline phosphatase* (*ALP*), *superoxide dismutase 1* (*SOD1*), *glutathione reductase* (*GR*), and *interleukin 10* (*IL-10*) targets, the observed Cq values, indicative of assay sensitivity, were mostly overlapping, and the specificity is optimal, as demonstrated by the melting curve plot. For all the genes, novel primers set was selected, because the ∆Rn values and parameter of amplification efficiency, were higher. Furthermore, the standard deviation (σ) of Cq between the two replicates was lower for the novel *SOD1* and *GR* systems. On the other hand, for the *IL12-β* gene, the system of Català et al., 2021 [23] was chosen for its best specificity as shown by the melting temperature plot. 

### 2.4. Differential Gene Expression Analysis Outcomes

**Figure 1:**
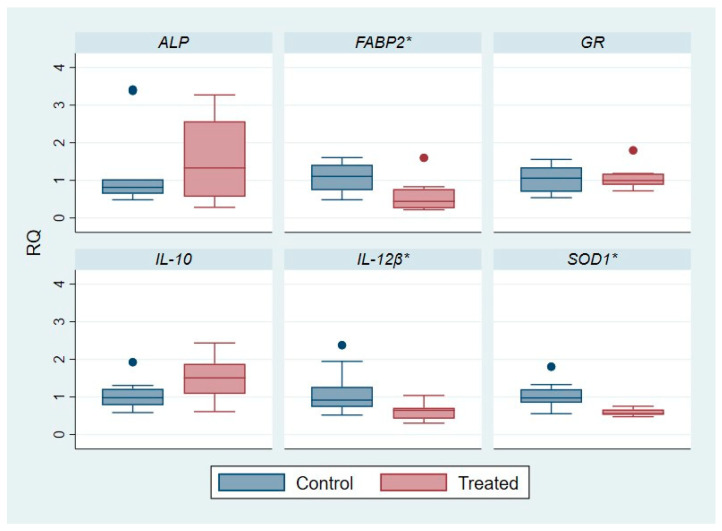
The effect of diet integrated with polyphenols on gene expression level (DGE) among control and treated group in fish livers. β-actin vas used as a housekeeping gene for normalization. RQ: relative quantification in comparative RTqPCR assay. *ALP: alkaline-phospatase*; *FABP2: fatty acid binding protein 2*; *GR: glutathione reductase*; *IL-10: interleukin-10*; *IL-12β: interleukin-12 subunit β*; *SOD1: superoxide-dismutase*. Dots: extreme values. *: *p*-value < 0.05.

**Figure 2:**
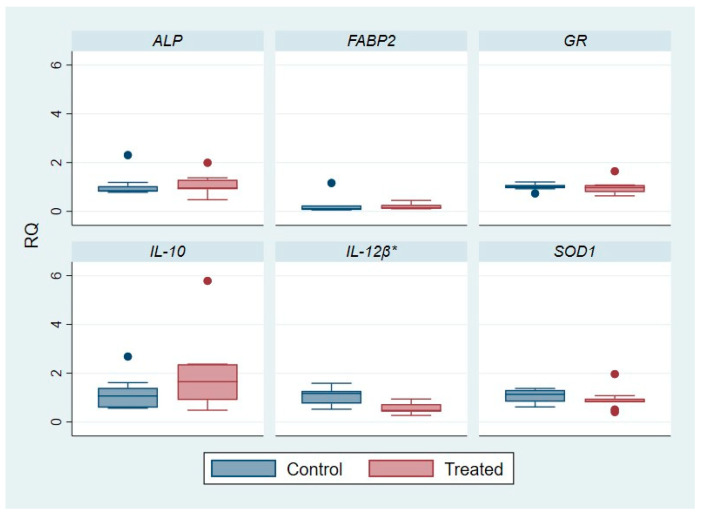
Effect of diet integrated with polyphenols on gene expression level (DGE) among control and treated group in fish posterior intestine. β-actin vas used as a housekeeping gene for normalization. RQ: relative quantification in comparative RTqPCR assay. *ALP: alkaline-phospatase*; *FABP2: fatty acid binding protein 2*; *GR: glutathione reductase*; *IL-10: interleukin-10*; *IL-12β: interleukin-12 subunit β*; *SOD1: superoxide-dismutase.* Dots: extreme values. *: *p*-value < 0.05.

The outcomes of Differential Gene Expression (DGE) analysis are reported in Supplementary Table S2 and in Figure 1 and Figure 2 where the relative quantification (RQ) parameter, indicative of relative mRNA/gene expression, was evaluated and compared.

Concerning the *ALP* gene, the gene expression level was maintained in both groups for the posterior intestine, while in the liver a slight fold change, in particular an up-regulation, was observed in the treated group. The treated mean RQ value was 1.58 and, thus, slightly higher than control mean RQ, but with a *p*-value not significant (*p*-value: 0.6497). Conversely, *FABP2* in the liver was significantly down-regulated in the treated group with a mean RQ value of 0.6 compared to the control one with a mean RQ of 1.07, and observing a significant *p*-value of 0.0308.

Both for the posterior intestine and for the liver, the *GR* gene was not differentially expressed (DE) in fish fed with standard feed compared to the subjects fed with polyphenol-based supplementation. On the other hand, for the *SOD1* gene, a down-regulation of gene expression was more marked in the liver of the treated group (mean RQ: 0.59) compared to the not treated one (mean RQ: 1.05), with a very significant *p*-value of 0.0022. For the SOD system, in the posterior intestine a similar trend on the gene level modulation was observed, but with a not significant (*p*-value: 0.4975) fold increase (mean RQ: 0.92 for treated one vs. mean RQ: 0.04 for control group).

Regarding the anti-inflammatory interleukin, *IL-10* gene, a slight over-expression was registered in the liver of treated subjects with a mean RQ of 1.45 compared to the control group with a mean RQ of 1.06, with a *p*-value of 0.1188.

In posterior intestine, a significant (*p*-value: 0.1845) trend of over-expression for *IL-10* was registered in specimens whose diet was integrated with polyphenols with a RQ mean of 1.96 compared to fish conventionally fed with a mean RQ of 1.15.

A significant down-regulation of the pro-inflammatory *IL-12β* gene expression was registered for the treated fish group, in the liver and in the posterior intestine, compared to the untreated fish. Specifically, for the liver, the mean RQ value was 0.63 for the treated group and 1.13 for the control one, with a significant *p*-value of 0.0433. Whereas at the posterior intestine the mean RQ value for *IL-12β* was 0.56 in the treated group and 1.06 in the control one, with a high significance level (*p*-value of 0.0025).

## 3. Discussion

As expected, in supplements and supplemented feeds, the predominant polyphenols are hydroxytyrosol and tyrosol, which are typical of the olive plant. However, it is interesting to note that in both feeds (control and supplemented) two other compounds with phenolic structure are also present at concentrations comparable to those of hydroxytyrosol and tyrosol, in the order of a few milligrams per kg (Table 1 and Table 2). These are vanillic acid and vanillin, which are not characteristic of olive trees, therefore, they certainly derive from the feed ingredients, also used during supplementation.

Fish fed with the experimental diet showed a tendency to increase in weight in the intermediate phase, while a higher weight increase was recorded at the end of the experiment and with a significant feed conversion ratio. A similar trend was observed for the specific growth rate (Supplementary Table S2). This result suggests a positive effect of dietary supplementation with polyphenols on fish general growth.

Other previous studies evaluated the implementation of olive oil bioactive compounds in fish diet. The findings of Rufino-Palomares et al., 2011 [25] demonstrated that maslinic acid as pentacyclic triterpene can stimulate the growth, white muscle protein turn-over rates and tissue hyperplasia in gilthead sea bream. Another study indicated that the feed intake with a certain percentage of olive oil bioactive extracts (polyphenols, triterpenic acids, long-chain fatty alcohols, unsaturated hydrocarbons, tocopherols, and sterols) increased the growth rate and affected the final *S. aurata* fish size distribution in body weight, making it more homogeneous [21].

Nonetheless, it can be deduced that the use in dietary polyphenols, derived from sustainable by-products in this case, determining an increase in weight (Supplementary Table S1), could reduce the effort for size selection during the farming and the production lots. Additionally, their utilization seems to maintain fish’s welfare parameter and a general good health status [24].

Even if the cross-talk of nutrition with immune system such as with other cellular pathways is well recognized, applied research about the interplay between diet and fish health has not be deepened as for mammalians species [26].

Gene expression level studies help to fully comprehend the effect of bioactive compounds in the diet on molecular and cellular pathways, regulating the responses to different diets, as well as in the present investigation (Figure 1 and Figure 2, Supplementary Table S2).

The anatomical districts here examined were the posterior intestine and liver. Specifically, the intestine is implicated not only in digestion and feed absorption but also in water and electrolyte balance, in the tracking of nutrients, and also in immunity, while the liver is particularly involved in metabolism and in response to oxidative stress [27]. Indeed, the target genes, here analyzed, play a crucial role in each investigated pathway, in fish. In particular, ALP is the alkaline phosphatase that can hydrolyze various phosphate compounds in intestines such as in other related body districts. The fatty acid binding protein 2 (FABP2), instead, is involved in intracellular lipid transport [28]. It binds free fatty acids and their coenzyme A derivatives, bilirubin, and some other small molecules in the cytoplasm mediating their trafficking in different districts.

Concerning ALP, it regulates the lipid absorption at enterocytes level with a contribution in the regulation of bicarbonate secretion and duodenal surface pH, protecting against the bacterial passage and the relative endotoxin-induced inflammation [21]. In the study of Gisbert et al., 2017 [21], in fish fed with olive oil bioactive compounds (OBE), the genetic up-regulation of *ALP* was observed, highlighting a higher enterocyte maturation as well as an improvement and enhancement of intestinal immune function. Additionally, the over-expression of the *ALP* gene could be dependent on their role in fatty acid uptake and trafficking. Their results further demonstrated that the OBE diet reduces the size of hepatic deposits within hepatocytes, determining lower lipid peroxidation values. Conversely, in our study, the *ALP* gene was not significantly differentially expressed between the control and polyphenol-treated group. While the values of RQ were uniformly distributed among the analyzed subjects for the posterior intestine, it was not the same for the liver district where the distribution was more variable in the treated group, as indicated also by the standard deviation (σ). Probably, differently from other olive oil bioactive compounds investigated in the literature [22], polyphenols are not implicated in this regulation route.

On the other hand, with regard to the gene modulation of *FABP2*, for the hepatic district it was significantly down-regulated in treated fish, with a *p*-value of 0.0308. Instead, in the posterior intestine, a significant fold modulation was not observed, even if the samples, as demonstrated by RQ values, were completely and equally distributed. The finding is in line with the general and common role of polyphenols, assumed with diet, as regulators of lipid metabolism [29]. Indeed, polyphenols can decrease the synthesis of fats and fatty acids in the liver and consequently their up-take and transport [30].

Glutathione reductase (GR) is implicated in the glutathione metabolic process, maintaining high levels of reduced glutathione in the cytosol and in the mitochondria, during the cell’s respiration/oxidative phosphorylation. It enables the protection of body cells including the blood component (particularly, the erythrocytes) from the oxidative stress: the active site is a redox-active disulfide bond, which regulates cell redox homeostasis [28]. Some oil and plant molecules have been proven able to inhibit the glutathione reductase in a concentration/dose- or time-dependent manner [31]. Some natural compounds have been demonstrated to regulate glutathione levels and function beyond their role as mere antioxidants. For example, certain compounds can up-regulate the expression of glutathione-related enzymes, increasing the availability of cysteine, limiting amino acid for glutathione synthesis, or directly interacting with its function modulation [32]. In our study, the *GR* gene was not differentially expressed (DE) in the treated group compared to control one both in the liver and in posterior intestine, rather the values were overlapping. Probably, we could assume that the polyphenolic molecules integrated to feeds are not completely implicated in the route glutathione (reductase) mediated or they do not exert any relevant effect at those concentrations or at those time of administration.

Additionally, SOD1 destroys radicals that are normally produced within the cells and that are toxic to biological systems [28]. On the other hand, the antioxidant mediators, also of aerobic organisms, are required to mitigate the negative effects and the damages of reactive oxygen and nitrogen species (RONS). These include SOD1, peroxidase, and catalase enzymes that play the function of RONS scavengers [33,34,35].

In the study of Naya-Català et al., 2021 [23], the effect of a fish diet based on processed animal proteins was evaluated. In particular, an up-regulation of hepatic *SOD1* expression was observed, pointing out, on the counterpart, a possible systemic cross-talk between fish antioxidant defense and the gut microbial community. On the other hand, the polyphenol-based diet given to our fish population was associated with a significant hepatic down-regulation of *SOD1* expression compared to the fish conventionally fed. The distribution of the RQ values was uniform among the analyzed subjects for the liver as well as for the posterior intestine, in which, however, a fold change between the groups was not observed. From these findings we could infer that the antioxidant properties of polyphenolic molecules themselves compensate for and partly limit the hepatic expression and, thus, the synthesis of endogenous SOD1 enzymes.

Concerning immunity and inflammatory mediators, interleukin 10 (IL-10) is a cytokine with immuno-modulatory activity and with predominant anti-inflammatory properties [28]. Instead, interleukin 12 (IL-12) is a cytokine with pro-inflammatory function. It activates the JAK/STAT signaling cascade, playing a role as a critical regulator of Th1-type immunity and promoting the production of other pro-inflammatory cytokines. Specifically, IL-12 is a heterodimeric cytokine characterized by p35 (IL-12α) and p40 (IL-12β) subunits [28]. In mammals, IL-12 is secreted by antigen presenting cells after activation by Pathogen Associated Molecular Patterns (PAMPs) and (Damage-associated Molecular Patterns (DAMPs), inducing NK cells to produce IFN-γ. Conversely, in fish, the presence of multiple divergent p40 subunits indicates that the IL-12 molecules could have different biological functions [36]. With regard to *IL-12β*, there are significant differences, in terms of DGE, between the two groups investigated in the present study. A significant down-regulation of expression was registered in treated subjects compared to the control ones, with a uniform distribution of samples, particularly for the posterior intestine district. The *p*-value registered was 0.0433 for liver and 0.0025 for the posterior intestine, underlying the anti-inflammatory properties of polyphenols introduced among the diet’s components. The outcomes of the present study are in accordance with literature data.

In particular, Estruch et al., 2018 [36], in the category of *S. aurata* fed with fish-meals (FM), observed an increase in gene expression in relation to inflammatory mediators related to the regulation of inflammation and the activation of innate immunity in response to the infection. In contrast, this up-regulation was not registered in the group fed with high plant protein-based diets (VM), which showed a lower expression of different pro-inflammatory markers and other genes linked to the immune defense (IGM, ALP) with a regulation of epithelial permeability. An important indicator of an anti-inflammatory profile in fish fed with a diet based particularly on vegetables proteins was the intestinal up-regulation of IL-10, a key anti-inflammatory cytokine used in both fish and mammals as an important marker for the health status of the host [23]. The same trend was observed in our study for the group of fish fed with polyphenols where an over-expression of the marker *IL10* was registered. The data were not statistically significant (*p*-value = 0.1188_liver; *p*-value = 0.1845_posterior intestine) but probably only for the presence of one extreme value in control group for the liver and one extreme value in each group for the posterior intestine (Figure 1 and Figure 2).

Future investigations on a broader panel of genes of immunity/inflammation, metabolic and oxidative stress pathways are also necessary in other fish model species. The goal is to confirm the current findings and properly characterize, by means of nutrigenomic, transcriptomic, or physiological approaches, the impact of polyphenols as well as of other olive oil derivatives in the health condition and metabolism of fish.

## 4. Materials and Methods

### 4.1. Diet Preparation and Formulation

The study was performed at a fish (*S. aurata*) farm located in the Tuscany region in Italy. Two different fish diets were formulated: (a) control diet and (b) diet supplemented (0.08%) with a polyphenol extract obtained from OMWW enriched with tyrosol and hydroxytyrosol. The extract, Stymon feed-10+, was provided by Stymon Natural Products P.C., Patras, Greece (www.stymon.com, last access on 9 September 2024). A preliminary trial was conducted to verify the more appropriate level of supplement to use in the experiment. This product derives from OMWW of *Olea europaea* L., Koroneiki cultivar. The extracts used were treated with hydrolytic enzymes, filtered by a membrane process, and powder encapsulated with a maltodextrin carrier (1:1 dw), followed by lyophilization (freeze-drying technique at −55 °C, 0.1 mbar) and a grinding process. The polyphenols extract was produced via a patented process (GR1010150, EP4049543A1) using green technologies. Feeds were prepared as a complete extruded fraction (diameter 4.5 mm) and analyzed by proximate composition according to standard methods [37]. Feed ingredients and chemical composition are indicated in Table 3. The experiment was carried out in compliance with the European animal safety guidelines used for research purposes according to the EU Directive 2010/63/EU, 22 September 2010 [38].

### 4.2. Polyphenols and Feed Analyses

Prior to the application of the protocol in the farm, the concentrations of polyphenols, both in the supplement (an extract derived from olive oil wastewater) and then in the feed, were analytically determined by means of liquid chromatography coupled to high resolution mass spectrometry.

#### 4.2.1. Preparation of Supplement Samples

About 1.0 g of supplement was weighed into a 50 mL Falcon^®^-type tube and 5 mL of the 80/20 (*v*/*v*) methanol/water extraction mixture containing 20 mg/L BHT was added. After 30 min of shaking and a centrifugation of 10 min, the supernatant was decanted into a 10 mL volumetric flask and the extraction was repeated. The pooled supernatants were diluted 1000-, 2500- and 5000-fold by taking 10 µL and placing them in a 10 mL, 25 mL and 50 mL flask, respectively; each flask was made up to volume with a mixture of 0.025% acetic acid/MeOH 90/10 (*v*/*v*). Quantitative analysis was performed by external standardization using a six-point calibration curve (0, 25, 50, 100, 250 and 500 ng/mL) prepared in a mixture of 0.025% acetic acid/MeOH 90/10 (*v*/*v*).

#### 4.2.2. Polyphenols Extraction and Preparation of Feed Samples

Polyphenol extraction of feed samples was carried out as reported by Branciari et al. in 2017 [39] with slight modifications. Briefly, 2.0 g of feed was weighed into a 50 mL Falcon^®^-type tube. One hundred microliters of hydroxytyrosol-d4 as Process Standard was added from a stock solution at 100 µg/mL, corresponding to an addition of 5 mg/kg. A total of 10 mL of the extraction mixture with MeOH/water 80/20 (*v*/*v*) containing 20 mg/L BHT was added and after 30 min shaking and 10 min centrifugation the supernatant was decanted into a 20 mL volumetric flask. Extraction under the same conditions was repeated for a second time. The pooled supernatants were diluted 5-fold by taking 1 mL of the extract, transferring it to a 5 mL volumetric flask and finally making it up to volume with a mixture of 0.025% acetic acid/MeOH 90/10 (*v*/*v*). Quantification was performed by external standardization using a matrix-matched calibration curve. In each analytical run, a “blank-sample” and the same sample fortified at 2.5 mg/kg with all analytes were analyzed as internal quality controls.

#### 4.2.3. Chemical Analysis and Instrumental Conditions

The polyphenols were chemically determined, and the included compounds were listed in Supplementary Table S3. The analysis was performed using an AB Sciex Exion LC^TM^ liquid chromatograph coupled to an AB Sciex 6600+ TripleTOF^TM^ mass spectrometer (AB Sciex, Framingham, MA, USA). Analytes were separated on an ACQUITY UPLC^®^ BEH C18 column (150 × 2.1 mm, 1.7 µm, Waters, Milford, MA, USA) equipped with an ACQUITY UPLC^®^ BEH C18 1.7 µm VanGuard™ pre-column (5 × 2.1 mm, Waters), using a flow rate of 250 µL/min and injection of 10 µL. The column temperature was maintained at 40 °C. The chromatographic gradient conditions are shown in Table S3. The analysis was performed in negative polarity (ESI-) and the acquisition in the MRM^HR^ (Multiple Reaction Monitoring) mode. The source conditions were as follows: the curtain gas was 40 psi, ion source GS1 and GS2 were set both at 55 psi, the ion-spray voltage was −4500 kV, and the capillary temperature was 450 °C. The monitored precursors and fragment ions with Declustering Potentials (DPs) and Collision Energies (CEs) are listed in Supplementary Table S4.

### 4.3. Field Study and Animals

A land-based facility in Central Italy, along the Tyrrhenian coast, where sea bass (*Dicentrarchus labrax*) and gilthead sea bream (*S. aurata*) are reared, was chosen for the field study.

The farm is located between the sea and a lagoon area and pumps water from the sea–lake connection channel, with a temperature between 6 °C and 30 °C depending on the season and a salinity of 38/40%. The system consists of 15 pre-fattening tanks with a volume of approximately 25 m^3^ each and 20 fattening tanks with a volume of 450 m^3^ each. The trial lasted 195 days (from 18 April 2022 to 29 October 2022) and it was performed in an indoor area of the farm using 6 cylindrical fiberglass tanks with a volume of approximately 6 m^3^ each, filled with a volume of water of approximately 3 m^3^. A saltwater recirculation system was used (2000 L of water per tank) with a rotating mechanical filter and a gravity bio-filter with a capacity of 36 m^3^. All tanks were equipped with aeration. The water temperature was 15 °C at the beginning of the experiment and reached 28–29 °C in July. The salinity of the water was 37.5, the dissolved oxygen was 6.50 ± 0.5 mg/L, and the pH varied between 7.5 and 8.5. The concentration of NH3 was <1 mg/L.

The experiment was conducted on a total of 600 gilthead sea bream, which were randomly assigned to two dietary groups (300 fish each). Each group was divided into three subgroups consisting of 100 fish placed in the six indoor tanks (Supplementary Figure S1).

The average initial/final weights for the control group were 238.2 ± 20.2/534.8 ± 64.3 and for the treated one were 215.4 ± 37.96/577.9 ± 66.37, respectively (Supplementary Table S1).

Fish were acclimatized in the tanks at the beginning of March 2022 and were all subjected to the same farm practices.

Natural photoperiod was maintained throughout the trial and all tanks were exposed to similar lighting conditions. In the tanks numbered 1, 2, and 3 (control group) the commercial feed was administered, while in the tanks numbered 4, 5, and 6 (supplemented group) the feed integrated with polyphenols was used. The chemical composition of the feed is shown in Table 3. Both diets (control and supplemented) used in the experiment were isonitrogenous [crude protein (CP): 45%] and isoenergetic (21 MJ kg^−1^ dry weight).

The fish were fed manually once a day, in the morning, and, to assess growth performance they were weighed individually at the start of the test (T0), after 84 days (T1), and after 195 days (T2), calculating the specific growth rate (% d − 1) = 100 × (ln final live weight − ln initial live weight/days). The calculated growth parameters are reported in Supplementary Table S1.

### 4.4. Fish General Health Assessment

The parameters of welfare and behavior, as well as the general health indexes, were assessed during the entire trial.

In particular, the main infectious pathogens were searched and detected by fresh microscopic examination of skin, gills, and intestinal contents; standard bacteriological examination of the kidney (sowing on blood and Marine agar plates, incubation in aerobiosis at 22 ± 1 °C for 24–48 h); end-point PCR for detection of encephalopathy-retinopathy virus (ERV); and histological examination for myxozoan enteroparasites (*Enteromyxum* spp.).

### 4.5. Sampling and Set Up for Gene Expression Study

In order to collect the organs and tissues of interest, the fish were first euthanized by overdose of anesthetic 3-aminobezeic acid ethyl ester (300–400 mg/L or 300–800 ppm) (MS-222, 1 g/L), in accordance with the good practices of fish welfare in experiments reported in Directive 2010/63/UE and in the Legislative Decree of 4 March 2014, n. 26 [38,40].

For the gene expression study, nine fish were sampled from each group (3 fish/tank) in order to conduct the analysis by means of RT-qPCR. In particular, for this trial, three specimens per three tanks (standard feed) and three specimens per three tanks (supplemented feed) were sampled.

Posterior intestine and liver were the selected tissues as targets for the inflammatory, oxidative stress, and metabolic pathways. The skin surface of the abdomen was cleaned with ethanol 70%, and a cut from the anus to the esophagus was made to remove the organs. For the subsequent gene expression analysis, tissue portions (at least 0.5 cm^3^) of the posterior intestine and of liver were put in at least 5 volumes of RNAlater (RiboSaver, GeneAll Biotechnology Co., Ltd.; Seoul, Republic of Korea) and then maintained for 24 h at 4 °C. The day after, samples were transferred into 2 mL cryovials, partly removing RNAlater liquid and then stored at −80 °C for the down-stream applications.

### 4.6. RNA Extraction and Quantification

RNA extraction was performed from about 60 mg of each collected intestinal and liver tissues homogenized in 1 mL of Trifast (VWR Company^®^, Avantor, Radnor Township, PA, USA), by TissueLyser II (Qiagen^®^, Hilden, Germany), with two steps at 20.0 Hz for 3′ and using 5 mm stainless steel beads. After the homogenization step, the RNA was purified, precipitated, and washed following the manufacturer’s instructions for the Trifast reagent (VWR Company^®^, Avantor). The extracted RNA from a total of 36 samples (9 posterior intestine and 9 livers of the control group; 9 posterior intestines and 9 livers of the treated group) was resuspended in 50 µL of nuclease free DEPC water. The RNAs’ quantity and quality were estimated with a BioSpectrometer fluorescence (Eppendorf^®^, Hamburg, Germany). RNA samples were also run on agarose gel to ensure the integrity and the absence of genomic DNA.

### 4.7. Gene Expression Analysis

The assay of gene expression was performed using SuperScript^TM^ III Platinum^TM^ SYBR^TM^ Green One-Step RT-qPCR Kit (Thermo Fisher Scientific, Waltham, MA, USA), an approach chosen to minimize acid nucleic handling or any contamination.

Target genes of inflammatory, metabolic, and oxidative stress pathways were selected on the basis of investigations conducted on *S. aurata*, where bioactives or other supplementation were integrated in fish feeds [21,22,23,24]. Except for the *β-actin* housekeeping gene, the oligonucleotide sets selected from the literature were compared with primer sets designed in this work using the Primer-BLAST software [41]. In the primer design we have also taken into account that the primer pair, for each transcript, was separated by at least one intron on the corresponding genomic DNA, thus, excluding any contaminations with the latter. By means of BLAST software [42], the sequences were aligned to evaluate the complete identity to the target, and the primers sequences were controlled for specificity in silico. Primer pairs were also chosen considering that their melting temperature was near to the annealing temperature at which the Taq enzyme works [43].

Detailed information about the gene primer sequences and references are reported in Table 4.

Extracted RNAs were diluted to about 1 µg/µL prior to the DNase digestion with DNase I, RNse-free (Thermo Fisher Scientific), in a 10 µL total volume, following the manufacturer’s instruction.

The target gene systems, from the literature and from the present study, were tested both for the fish posterior intestine and for liver tissues.

For each target, RT-qPCR assays were tested for specificity and efficiency. Then, the assays were optimized at the same conditions as follows: 25 µL of reaction volume, 0.5 µL of SuperScript™ III RT/Platinum™ Taq Mix (Thermo Fisher Scientific), 12.5 µL of SYBR Green Reaction Mix, 400 nM of forward primer and reverse primer, ROX reference dye (diluted 1:10), DEPC treated water to adjust the volume, and 5 µL of RNA; thus, in total, an estimated concentration of 500 ng. Quantitative PCR (qPCR) reactions were performed using a QuantStudio™ 7 Flex Real-Time PCR System (Thermo Fisher Scientific, Applied Biosystems^TM^) with the following thermal profile: a hold stage at 50 °C × 5′ for the retro-transcription step of mRNA in cDNA, another hold stage at 95 °C × 2′, and 40 cycles of amplification step in Fast mode at 95 °C × 3″ and 60 °C × 30″.

Melting curve stages consist of a first step at 95 °C × 15″, a second step at 60 °C × 1′, and a third dissociation step at 95 °C × 15″.

Each sample was tested both for β-actin and for the specific target gene. For all the samples, each system versus the endogenous gene was assessed in the same working session to ensure reproducibility and to mitigate the variability on the gene expression level modulation. Fluorescence data acquired during the extension phase were determined with the comparative ∆∆Cq method [44] using β-actin as housekeeping gene and as normalizer of any over-estimations and any fluctuations not depending on gene expression level. β-actin was used because of its major stability under different experimental conditions [20,21,22,23].

Negative controls, with nuclease free DEPC water instead of RNA, were introduced in each working session.

### 4.8. Statistical Analysis and Elaboration

QuantStudio™ 7 Software data were elaborated, DGE analysis was carried out [42], and differences between groups (treated and control) were evaluated using Student’s *t*-test for each gene and each matrix.

The significance level has been set at 0.05.

Microsoft Excel 2013 and Stata Statistical Software: Release 16 were used to conduct all the analyses.

## 5. Conclusions

The outcomes of the present study suggested that dietary supplementation with OMWW polyphenols can enhance fish growth while maintaining the welfare and the general health fish conditions. Interestingly, as demonstrated by gene expression data, the polyphenolic supplementation exerted positive effects not only on the innate immunity but also on the oxidative stress and the metabolic pathways with more evidence at the hepatic level when compared to the intestinal one. These results highlighted that polyphenols, derived from OMWW, represent high-value biomolecules, confirming the scarce number of data present in the literature on the topic [45,46,47]. Thus, these bioactive compounds could be used and labeled as novel ingredients, being a promising feed additive for the aquaculture industry with multiple beneficial properties also in a sustainability direction and in a circular economy perspective. However, further experiments on a broader panel of genes are necessary to confirm or implement these data in gilthead seabream. In addition, other aspects should be in depth investigated in the future, to better clarify the properties and the benefits of polyphenols derived from these olive oil by-products and, thus, their re-use in the fish diet and, generally, in animal feeding.

## Figures and Tables

**Table 1:** Concentration (mg/g) of the detected analytes in the polyphenol extract used in the supplemented diet.

ID	Analyte	Concentration (mg/g)
StymonFeed-10+	Hydroxytyrosol	2.6
	Tyrosol	0.5
	Vanillic acid	0.11
	Vanillin	0.01
	p-Coumaric acid	0.03
	Verbascoside	0.06
	Oleacein	0.05
	Oleuropein	0.04
	Pinoresinol	0.01
	Oleuropein aglycone	0.02
	Luteolin	0.76
	Apigenin	0.06
	**Total polyphenols**	**4.30**

**Table 2:** Concentration (mg/kg) of the detected analytes compared to the total polyphenol (mg/kg) in pre-supplemented control feed and in the final feeds.

ID	Analyte	Concentration (mg/kg)	Total Polyphenol (mg/kg)
Control feed	Tyrosol	3.4	12.4
	Vanillic acid	7.3	
	Vanillin	1.7	
0.08% StymonFeed 10+	Hydroxytyrosol	7.0	21.1
	Tyrosol	4.2	
	Vanillic acid	6.6	
	Vanillin	1.5	
	Verbascoside	0.4	
	Luteolin	1.1	
	Apigenin	0.3	

**Table 3:** Formulation and proximate composition of the diet.

Ingredients (g/kg)	
Fish meal	248
Maize gluten meal	290
Dehulled sunflower meal	180
Fish oil	110
Wheat	105
Wheat flour	50
Calcium carbonate	5
Mineral premix	5
Vitamin premix	5
Technological additives	2
**Proximate composition (g/kg as fed)**	
Moisture	85.0
Crude protein	450.0
Crude lipids	160.0
Crude fiber	26.0
Ash	81.0
Calcium	15.0
Phosphorus	11.0
Sodium	2.2
Gross energy (MJ/kg DM)	21.0

**Table 4:** Information about the targets investigated in the gene expression pathways by means of RT-qPCR.

Pathway	Target Gene	Symbol	Primer Sequence (5′-3′)		GeneBank (Reference Sequence)	Product Lenght	References
housekeeping reference gene	*beta-actin*	***β-Act***	For	TCCTGCGGAATCCATGAGA	X89920	51 bp	[20,21,23]
			Rev	GACGTCGCACTTCATGATGCT			
immune-inflammatory	*interleukin-12 subunit beta*	***IL12-β***	For	ATTCCCTGTGTGGTGGCTGCT	JX976624	54 bp	[23]
			Rev	GCTGGCATCCTGGCACTGAAT			
		***IL12-β-n***	For	GGTAGTTTGGGAGTGTGTCTGA		92 bp	This study
			Rev	GGAACTTGGTGTGTGGGATTT			
	*interleukin-10*	***IL10***	For	AACATCCTGGGCTTCTATCTG	JX976621	119 bp	[23]
			Rev	GTGTCCTCCGTCTCATCTG			
		***IL10-n***	For	CGAGACTTCTACGAAGCAAACG		86 bp	This study
			Rev	CAGGCGAACGCTGTTTTGAA			
oxidative stress	*superoxide dismutase* *[Cu-Zn]*	***SOD1***	For	TCACGGACAAGATGCTCACTCTC	JQ308832	53 bp	[20]
			Rev	GGTTCTGCCAATGATGGACAAGG			
		***SOD1-n***	For	GGGTCGTTCATTTTGAGCAGG		76 bp	This study
			Rev	CCGGGAGTAAGCCCTTTGATT			
	*glutathione reductase*	***GR***	For	TGTTCAGCCACCCACCCATCGG	AJ937873 (XM_030396244.1 *)	115 bp	[20]
			Rev	GCGTGATACATCGGAGTGAATGAAGTCTTG			
		***GR-n***	For	GTCGGTGTCACAGGCACTAC		100 bp	This study
			Rev	GATCACCAGAAAGTCGAACCG			
metabolic	*fatty acid-binding protein*	***FABP2***	For	CGAGCACATTCCGCACCAAAG	KF857310	93 bp	[23]
			Rev	CCCACGCACCCGAGACTTC			
		***FABP2-n***	For	CCGGAAAGACAACAGCAAGC		92 bp	This study
			Rev	TAGCGTCCACTCCCTCATAGT			
	*alkaline phosphatase*	***ALP***	For	CCGCTATGAGTTGGACCGTGAT	KF857309	63 bp	[23]
			Rev	GCTTTCTCCACCATCTCAGTAAGGG			
		***ALP-n***	For	TGCCGTGACATTGCATACCA		70 bp	This study
			Rev	ACTGACGACCTCCACCTAGA			

* variant X2; mitochondrial isoform mRNA; bp: base pair; For: forward primer; Rev: reverse primer; n: new primers set, designed in this study

## Data Availability

Data is contained within the article (and Supplementary Materials).

## Supplementary Materials

The following supporting information can be downloaded at: https://www.mdpi.com/article/10.3390/ijms252212185/s1.
