# Supplementary material for: Gene Expression Study in Gilthead Seabream (Sparus aurata): Effects of Dietary Supplementation with Olive Oil Polyphenols on Immunity, Metabolic, and Oxidative Stress Pathways
Source: Int J Mol Sci. 2024 Nov 13;25(22):12185. doi: 10.3390/ijms252212185 (PMC11594698; doi:10.3390/ijms252212185)

International Journal of *Molecular Sciences*

MDPI

Table S1. Growth parameters of control group and of group treated with the supplemented diet (average measures with standard deviations and p-value)

	Control Group	Treated group	p-value
Initial weight (gr)	238.2±20.2	215.4±37.96	0.15
Final weight (gr)	534.8±64.3	577.9±66.37	0.26
Final weight gain (%)	124.7±30.1	176.9±64.9	0.04
Specific growth rate	0.40±0.07	0.50±0.18	0.05
Feed conversion ratio	1.85±0.13	1.45±0.05	0.02

Table S2. DGE (differential gene expression) analysis: gene expression difference between control and treated group for liver and posterior intestine, with mean, standard deviation, confidence interval 95% min, max and p-value of the Student's t-test.

gene	matrix	**RQ mean_control**	ds	ci-	ci+	min	max	**RQ mean_treated**	ds	ci-	ci+	min	max	p-value (ttest)
ALP	liver	1.32	1.19	0.4	2.23	0.483	3.413	1.58	1.18	0.67	2.48	0.282	3.27	0.6497
ALP	posterior intestine	1.07	0.49	0.69	1.44	0.781	2.307	1.05	0.46	0.69	1.41	0.477	1.993	0.945
FABP2	**liver**	**1.07**	**0.39**	**0.77**	**1.37**	0.484	1.604	**0.6**	**0.44**	**0.26**	**0.94**	0.218	1.598	**0.0308**
FABP2	posterior intestine	0.27	0.4	0	0.64	0.057	1.162	0.19	0.13	0.08	0.31	0.104	0.448	0.6231
GR	liver	1.06	0.37	0.78	1.34	0.539	1.556	1.07	0.32	0.83	1.32	0.72	1.797	0.9495
GR	posterior intestine	1.01	0.14	0.9	1.12	0.733	1.204	1	0.29	0.78	1.22	0.636	1.645	0.9275
SOD1	**liver**	**1.05**	**0.37**	**0.77**	**1.33**	0.555	1.805	**0.59**	**0.1**	**0.51**	**0.67**	0.475	0.753	**0.0022**
SOD1	posterior intestine	1.04	0.29	0.82	1.26	0.616	1.375	0.92	0.44	0.57	1.26	0.407	1.964	0.4975
IL10	liver	1.06	0.4	0.75	1.36	0.585	1.925	1.45	0.6	0.99	1.91	0.611	2.435	0.1188
IL10	posterior intestine	1.15	0.7	0.62	1.69	0.563	2.682	1.96	1.59	0.73	3.18	0.482	5.787	0.1845
IL12β	**liver**	**1.13**	**0.63**	**0.64**	**1.61**	0.519	2.378	**0.63**	**0.26**	**0.43**	**0.83**	0.303	1.041	**0.0433**
IL12β	**posterior intestine**	**1.06**	**0.35**	**0.79**	**1.33**	0.523	1.588	**0.56**	**0.23**	**0.39**	**0.74**	0.268	0.941	**0.0025**

Table S3. Retention times (RT) and monitored ions of the polyphenols determined in the study.

	Analyte	RT (min)	Adduct	Molecular formula	Precursor ion (*m/z*)	Q-ion (quantifier) (*m/z*)	q-ion (qualifier) (*m/z*)	DP (V)	CE (V)
1	Hydroxytyrosol	9.2	$[M-H]^-$	$C_8H_{10}O_3$	153.1	123.0455	153.0583	-80	-14
	Hydroxytyrosol-d4[a]	9.2	$[M-H]^-$	$C_8H_6D_4O_3$	157.1	125.0588	-	-80	-15
2	Tyrosol	11.9	$[M-H]^-$	$C_8H_{10}O_2$	137.1	119.0520	119.0520	-90	-18
3	Vanillic acid	13.3	$[M-H]^-$	$C_8H_8O_4$	167.0	152.0111	167.0347	-70	-15
4	Vanillin	14.9	$[M-H]^-$	$C_8H_8O_3$	151.0	136.0166	151.0401	-60	-14
5	Verbascoside	16.4	$[M-H]^-$	$C_{29}H_{36}O_{15}$	623.1	161.0251	623.2066	-90	-38
6	Oleuropein	17.3	$[M-H]^-$	$C_{25}H_{32}O_{13}$	539.1	307.0824	539.1807	-100	-27
7	Pinoresinol	17.4	$[M-H]^-$	$C_{20}H_{22}O_6$	357.1	151.0410	357.1396	-80	-20
8	Oleacein (3,4 DHPEA-EDA)	17.4	$[M-H]^-$	$C_{17}H_{20}O_6$	319.1	195.0663	319.1177	-100	36
9	Luteolin	17.5	$[M-H]^-$	$C_{15}H_{10}O_6$	285.0	133.0293	285.0405	-110	-36
10	Oleuropein aglycon aglygone	17.6	$[M-H]^-$	$C_{19}H_{22}O_8$	377.1	307.0824	377.1239	-80	-14
11	Apigenin	17.7	$[M-H]^-$	$C_{15}H_{10}O_5$	269.0	117.0343	269.0451	-110	-35

[a]Hydroxytyrosol-d4 was used to check recovery rates of analytes in feed samples.

Table S4. Cromatographic conditions for polyphenols chemically characterization**.**

Time (min)	% Mobile phase A (acetic acid 0.025% in H_2O)	% Mobile phase B (MeOH/ACN 90:10)
0	100	0
1	100	0
11	80	20
15	50	50
16	0	100
20	0	100
21	100	0
26	100	0

Figure S1. Some *Sparus aurata* specimens in one of the experimental tanks.

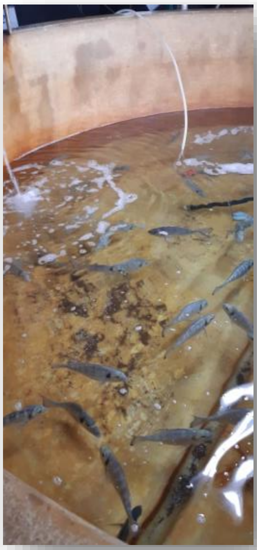

Supplement: Supplementary file 1 [file ijms-25-12185-s001.zip › ijms-3240683-supplementary.pdf]
